# Supplementary material for: Predicting visual function by interpreting a neuronal wiring diagram
Source: Nature. 2024 Oct 2;634(8032):113–23. doi: 10.1038/s41586-024-07953-5 (PMC11446822; doi:10.1038/s41586-024-07953-5)
Supplement: Supplementary file 6 — CRF and ERF predictions for individual TmY4 and TmY9 cells. Analogous to Supplementary Data 3, but for TmY target types. Shown are the top four monosynaptic pathways, the strongest pathway passing through each of the top ten intermediary types (ranking from Extended Data Fig. 7), and the trisynaptic pathway Tm1–TmY–Dm3–TmY (see the section entitled Prediction of spatial normalization). [file 41586_2024_7953_MOESM6_ESM.zip › DataS4/TmY9qΓèÑ/720575940630678902.pdf]

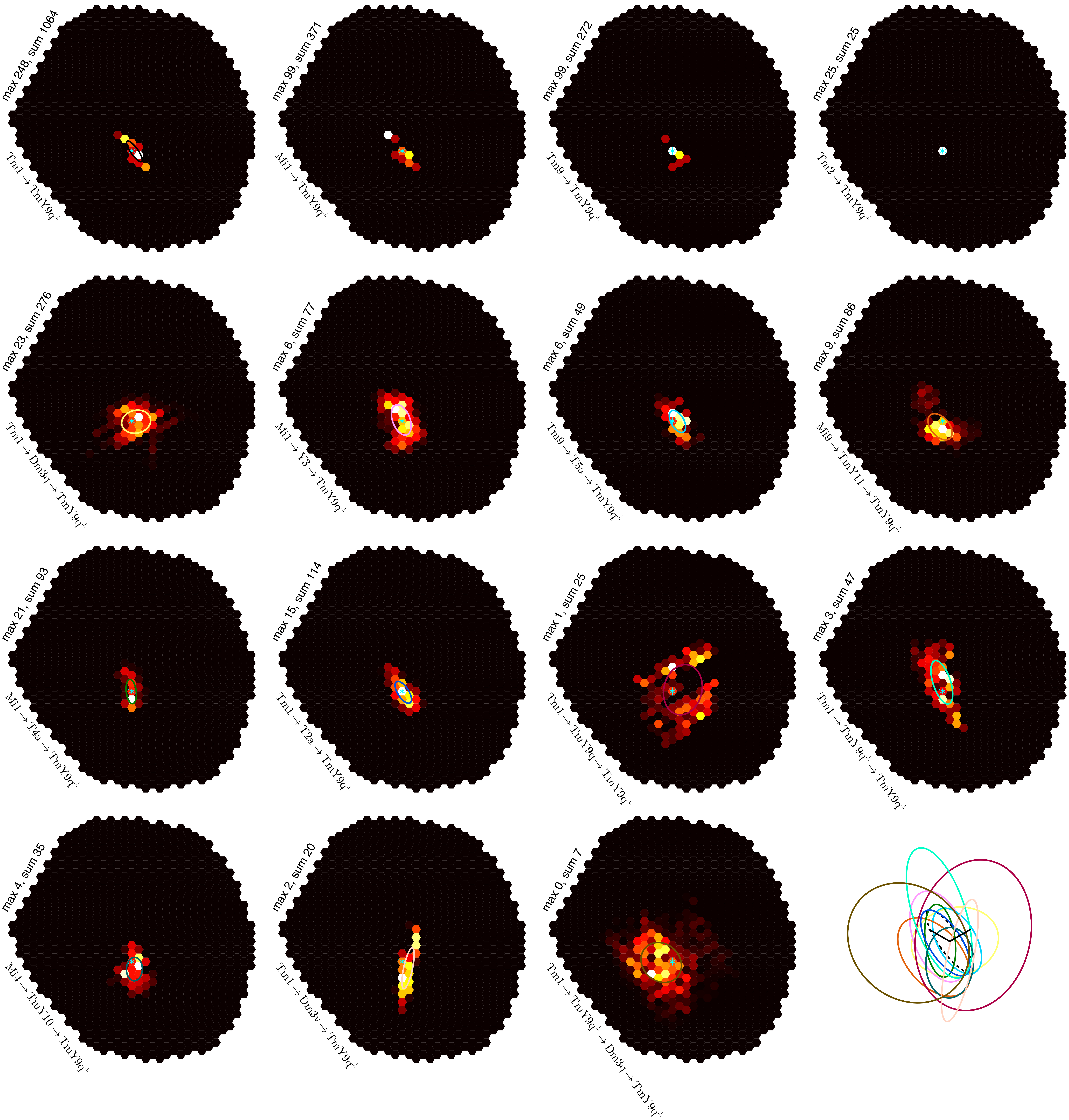

max 248, sum 1064
Tm1 → TmY9q⊥
max 99, sum 371
Mi1 → TmY9q⊥
max 99, sum 272
Tm9 → TmY9q⊥
max 25, sum 25
Tm2 → TmY9q⊥
max 23, sum 276
Tm1 → Dm3q → TmY9q⊥
max 6, sum 77
Mi1 → Y3 → TmY9q⊥
max 6, sum 49
Tm9 → T5a → TmY9q⊥
max 9, sum 86
Mi9 → TmY11 → TmY9q⊥
max 21, sum 93
Mi1 → T4a → TmY9q⊥
max 15, sum 114
Tm1 → T2a → TmY9q⊥
max 1, sum 25
Tm1 → TmY9q → TmY9q⊥
max 3, sum 47
Tm1 → TmY9q⊥ → TmY9q⊥
max 4, sum 35
Mi4 → TmY10 → TmY9q⊥
max 2, sum 20
Tm1 → Dm3v → TmY9q⊥
max 0, sum 7
Tm1 → TmY9q⊥ → Dm3q → TmY9q⊥
